# Suppression of MAPK attenuates neuronal cell death induced by activated glia-conditioned medium in alpha-synuclein overexpressing SH-SY5Y cells

**DOI:** 10.1186/s12974-015-0412-7

**Published:** 2015-10-26

**Authors:** Lidia M. Yshii, Alexandre Denadai-Souza, Andrea R. Vasconcelos, Maria Christina W. Avellar, Cristoforo Scavone

**Affiliations:** Department of Pharmacology, Molecular Neuropharmacology Laboratory, Institute of Biomedical Science ICB-1, University of São Paulo, Avenida Professor Lineu Prestes, 1524, São Paulo, 05508-900 Brazil; Department of Pharmacology, Section of Experimental Endocrinology, Federal University of São Paulo, São Paulo, Brazil

**Keywords:** α-Synuclein, MAPK, NF-κB, Cell death

## Abstract

**Background:**

Parkinson’s disease (PD) is a neurodegenerative disease with characteristics and symptoms that are well defined. Nevertheless, its aetiology remains unknown. PD is characterized by the presence of Lewy bodies inside neurons. α-Synuclein (α-syn) is a soluble protein present in the pre-synaptic terminal of neurons. Evidence suggests that α-syn has a fundamental role in PD pathogenesis, given that it is an important component of Lewy bodies localized in the dopaminergic neurons of PD patients.

**Methods:**

In the present study, we investigated the influence of wild type (WT) and A30P α-syn overexpression on neuroblastoma SH-SY5Y toxicity induced by the conditioned medium (CM) from primary cultures of glia challenged with lipopolysaccharide (LPS) from *Escherichia coli*.

**Results:**

We observed that SH-SY5Y cells transduced with α-syn (WT or A30P) and treated with CM from LPS-activated glia cells show evidence of cell death, which is not reverted by NF-κB inhibition by sodium salicylate or by blockage of P50 (NF-κB subunit). Furthermore, the expression of A30P α-syn in neuroblastoma SH-SY5Y decreases the cell death triggered by the CM of activated glia versus WT α-syn or control group. This effect of A30P α-syn may be due to the low MAPK42/44 phosphorylation. This finding is substantiated by MEK1 inhibition by PD98059, decreasing LDH release by CM in SH-SY5Y cells.

**Conclusion:**

Our results suggest that SH-SY5Y cells transduced with α-syn (WT or A30P) and treated with CM from LPS-activated glia cells show cell death, which is not reverted by NF-κB blockage. Additionally, the expression of A30P α-syn on neuroblastoma SH-SY5Y leads to decreased cell death triggered by the CM of activated glia, when compared to WT α-syn or control group. The mechanism underlying this process remains to be completely elucidated, but the present data suggest that MAPK42/44 phosphorylation plays an important role in this process.

**Trial Registration:**

PROSPERO: CRD42015020829

## Background

Parkinson’s disease (PD) is the most common neurodegenerative movement disorder [1]. The clinical symptoms include rigidity, slowness of movement and tremor [2, 3]. The pathological hallmark of PD is dopaminergic neuronal (DN) loss in the substantia nigra and the presence of intracellular inclusions called Lewy bodies, largely composed of aggregated α-synuclein (α-syn) [4, 5]. The physiological role of α-syn is unknown. However, recent data supports its role as a synaptic protein involved in neurotransmitter vesicular release [6, 7]. Multiplications of the human α-syn gene or point mutations (A30P, A53K, E46K) are related to familial autosomal-dominant forms of early-onset PD [8–12]. This suggests that α-syn overexpression or point mutations may accelerate disease onset and progression. Moreover, genome-wide association studies have identified *SNCA*, the gene that encodes α-syn, as having a strong association with the development of PD [13].

The inflammatory component in PD is reinforced by studies showing high microglia concentration and increased release of cytokines in the brains of PD patients [14]. Furthermore, the chronic inflammation in PD that contributes to DN degeneration is maintained by activated microglia and astrocytes, as well as T lymphocyte infiltration from the periphery [15–17]. Activated microglia release proinflammatory mediators that can amplify the inflammatory response, producing a deleterious effect in DN [18–20].

The mechanisms underlying the loss of DN in PD are still unclear. The nuclear factor-kappa-light-chain-enhancer of activated B cells (NF-κB) is a transcription factor that regulates gene expression in numerous processes, including inflammation and apoptosis [21–23]. NF-κB can form homodimers or heterodimers from the NF-κB family of proteins p65 (RelA), p50 (NF-κB1), RelB, cRel and p52 (NF-κB2). The inactive form of NF-κB is kept in the cytosol. Following stimulation, the inhibitor of κB (IκB) protein is phosphorylated, permitting NF-κB to translocate to the nucleus. NF-κB is activated in the brains of PD [24] and Alzheimer’s disease patients [25, 26], contributing to the pathological progression of these neurodegenerative diseases [27]. In this study, we investigated the NF-κB response in neuroblastoma cells (SH-SY5Y) overexpressing wild type (WT) or A30P α-syn, which have been challenged with the conditioned medium (CM) of lipopolysaccharide (LPS)-stimulated primary glia cultures. We observed that the treatment of the WT or A30P α-syn overexpressing cells with CM increased cell death, although the inhibition of NF-κB did not affect cell death levels. We further demonstrate that this CM-induced cell death is abrogated by the inhibition of MEK1 (MAPK/extracellular signal-regulated kinase kinase 1).

## Methods

### Lentiviral vectors

cDNA coding for WT or A30P human α-syn GenBank no. NM_000345.2 (kindly provided by Dr. Pamela McLean, Harvard University, USA) was cloned in the pHRIG lentiviral vector (a generous gift from Dr. Robert Sapolsky, Stanford University, USA). The packaging construct and vesicular stomatitis virus G protein envelope used were the pMDL, pREV and pVSVG plasmids (Invitrogen). The viral particles were produced by transfection of HEK 293 T cells with these plasmids [28].

### Primary cell culture

Primary mixed glial cultures were prepared from the cortex of 1–3-day-old Wistar rats as described [29]. Briefly, cortices free of meninges were digested with Ca^2+^- and Mg^2+^-free HBSS (Invitrogen) containing 0.05 % trypsin and 10 μg/mL DNase (Sigma) and triturated with DMEM (Invitrogen) containing 10 % heat-inactivated foetal bovine serum (FBS, Invitrogen) and 1 % penicillin-streptomycin. Dissociated cells were plated onto 75-cm^2^ flasks and fed every other day for 14 days. On the 15th day, the cells were treated with LPS (*Escherichia coli* 0111:B4, Sigma) 1 μg/mL in DMEM without FBS for 24 h. The supernatants were collected and named conditioned medium (CM). All the experimental procedures were performed in accordance with the standards of the Ethics Committee on Animal Experimentation of the Institute of Biomedical Sciences, University of São Paulo (ICB-USP) and followed all the requirements described in the Brazilian College of Animal Experimentation (COBEA) in compliance with the National Institutes of Health guide for the care and use of laboratory animals. Moreover, all efforts were made to minimize animal suffering and to reduce the number of animals used.

### Cell culture

SH-SY5Y neuroblastoma cells (CRL-2266, ATCC) were maintained in DMEM supplemented with 10 % FBS, 100 Units/mL penicillin and 0.1 mg/mL streptomycin in humidified 5 % CO_2_ atmosphere at 37 °C. For each experiment, 1 × 10^5^ cells were transduced with 1 × 10^4^ infectious units per volume of lentivirus containing empty pHRIG, pHRWT α-synIG or pHRA30 [30]. Experiments were performed in 6-well or 96-well culture plates at 80 % cell confluence. SH-SY5Y cells were treated with CM, PD98059 (Millipore), peptide SN50/SN50M (Calbiochem) or sodium salicylate (NaSal, Sigma) for 24 h in DMEM with 0.1 % FBS.

### Measurement of TNF-α, IL-1β and KC

The CM of mixed glial culture was collected and concentrations of tumour necrosis factor-alpha (TNF-α) and interleukin (IL)-1β were measured using commercial ELISA kits (R&D Systems), following the manufacturer’s instructions. The detection limit of the method is 10.9 (minimum) and 700 pg/mL (maximum).

### Lactate dehydrogenase release assay

As described previously [31], cell viability was evaluated by lactate dehydrogenase (LDH) release into the cell culture supernatants by enzymatic test, using the Cytotox 96 kit (Promega). In brief, a volume of 50 μL of culture medium was transferred to a 96-well microplate, into which a 50 μL of substrate mix was added and incubated for 30 min at room temperature, followed by 50 μL of stop solution (1 M acetic acid). The optical density at 490 nm was measured using a microplate reader (Biochrom). The percentage of cell death was normalized to the LDH values released after exposing cells for 45 min to lysis solution (9 % Triton X-100), which was expressed as 100 % cell death.

### Electrophoretic mobility shift assay (EMSA)

Nuclear extracts from control or treated SH-SY5Y cells were prepared as previously described [32]. Double-stranded oligonucleotide containing the NF-κB consensus sequence from Promega (5′-AGTTGAGGGGACTTTCCCAGGC-3′) was end labelled using T4 polynucleotide kinase (Promega) in the presence of γ-^32^P dATP. Nuclear extracts (5 μg) were incubated with ^32^P-labelled NF-κB probe. The binding reaction was performed at room temperature for 30 min in a reaction buffer containing 50 mM Tris-HCl pH 7.5, 250 mM NaCl, 5 mM MgCl_2_, 2.5 mM EDTA, 20 % glycerol, 0.25 μg/μL of poly (dI-dC) and 2.5 mM dithiothreitol. DNA protein complexes were separated by electrophoresis through a 6 % acrylamide:bis-acrylamide (37.5:1) gel in TBE (45 mM Tris, 45 mM boric acid, 0.5 mM EDTA) for 2 h at 150 V. Gels were vacuum dried for 1 h at 80 °C and exposed to X-ray film at −80 °C. For competition assays, 5 μg of nuclear extract was incubated with specific competitor (unlabelled double-stranded NF-κB consensus oligonucleotide) or a non-specific competitor (unlabelled transcription initiation factor IID [TFIID]). For supershift assay, antibodies against subunits of NF-κB (p50, p65, cRel, RelB 1:20) (Santa Cruz Biotechnology) were added into the binding reactions.

### Western blot analysis

Cells were homogenized in lysis buffer (10 mM HEPES pH 7.9, 1.5 mM MgCl_2_, 10 mM KCl, 0.1 mM EDTA, 30 mM NaF, 3 mM orthovanadate, 0.5 mM DTT, 2 mM sodium pyrophosphate, 0.5 mM PMSF, 2 μg/mL leupeptin, 2 μg/mL antipain) and incubated on ice for 10 min. After addition of NP-40 (0.5 %), samples were vigorously mixed and centrifuged for 30 s at 12,000 × *g*, and the resulting supernatant was collected. Protein concentration in cell lysates was determined using a protein assay kit (Bio-Rad). The immunoblotting was performed as described previously [26]. The primary antibodies used were the following: α-synuclein (1:500, BD Transduction Laboratories), phospho-MAPK42/44 (1:2000, Cell Signalling Technology), α-tubulin (1:2000, Santa Cruz Biotechnology) and β-actin (1:2000, Sigma). The HRP-conjugated secondary antibodies (1:5000, Sigma) were applied and blots were developed using an ECL kit (Millipore).

### Immunofluorescence

The immunofluorescence was performed as described previously [26] , with some modifications. Cells were fixed in cold 4 % formaldehyde for 15 min before incubation in blocking solution (5 % normal donkey serum/PBS 0.01 % Triton X-100). Cells were incubated with rabbit anti-GFAP antibody (1:500; Millipore), mouse anti-MAP2 antibody (1:500; Abcam), mouse anti-CD68 (1:200; Abcam) or anti-TNFR1 (1:300; Abcam) overnight. After washing in PBS, cells were incubated with (1:1000) anti-rabbit Alexa Fluor 488 and anti-mouse Alexa Fluor 594. DAPI was used to stain nuclei. Control cells were incubated only with secondary antibodies. Images were analysed under a fluorescence microscope (Nikon Eclipse 80i) or confocal microscope (Zeiss LSM 780).

## Results

### Cells treated with CM release LDH in dose-dependent way

**Fig. 1:**
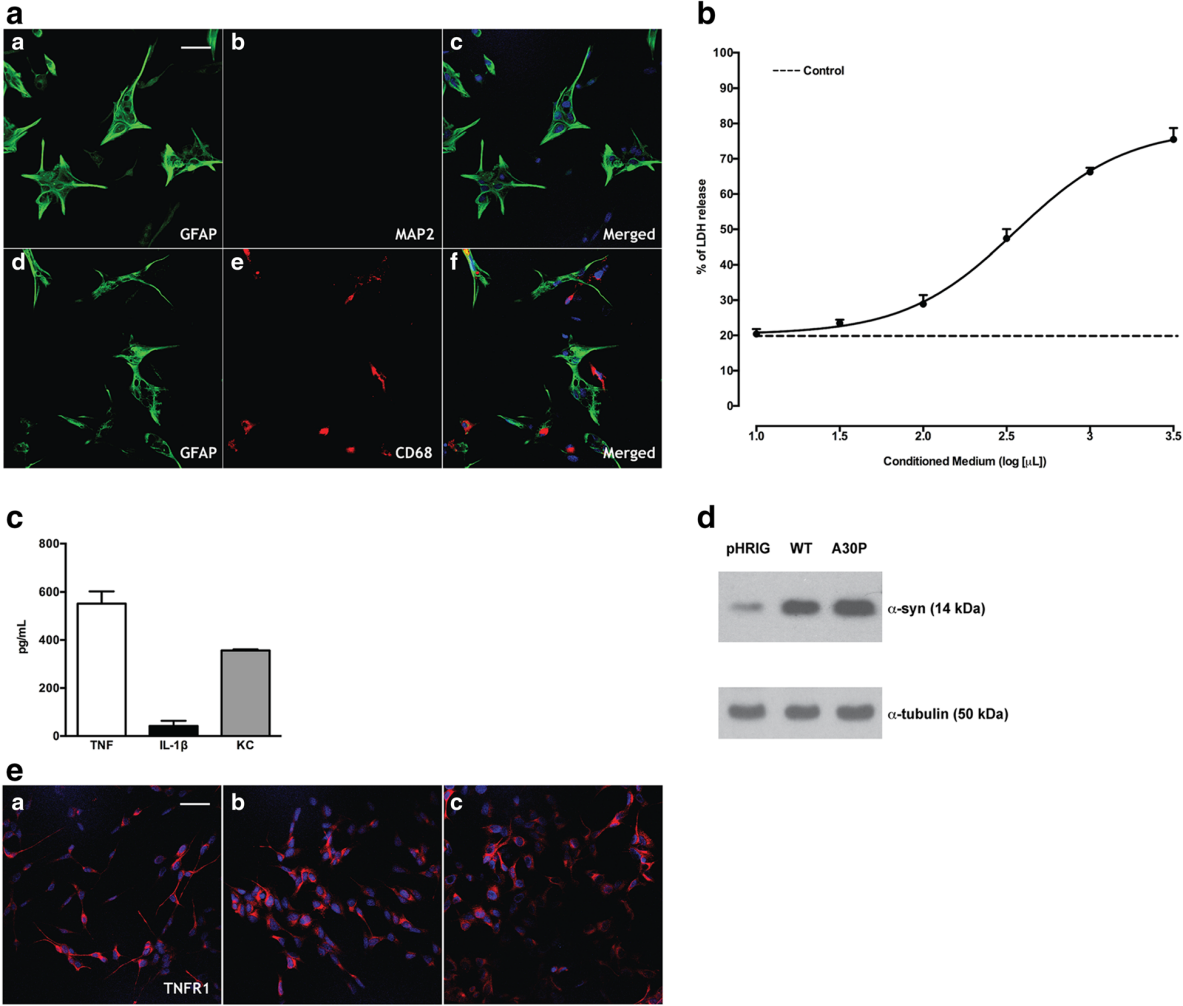
Characterization of primary glial culture, conditioned medium and overexpression of α-syn. **a** Immunofluorescence assay was performed in primary glial culture of rat cortex using (*A*) GFAP (*green*), (*B*) MAP2 (*red*) or (*E*) CD68 (*red*) antibodies. Glial cells are stained with glial-specific glial fibrillary acidic protein (GFAP) or CD68 (microglia) but not with MAP2. The nuclei are stained with DAPI. Scale bar: 50 μm. **b** LDH liberation by SH-SY5Y cells treated with CM. Dose-dependent increase of LDH liberation after treatment with CM on SH-SY5Y cells. The *dotted line* represents the cells treated with PBS (19.45 % ± 0.98; *n* = 6). **c** Production of inflammatory cytokines or chemokine by activated glia. No detectable quantity of TNF-α, IL-1β or KC was detected in CM of non-activated glia. The values represent the mean ± SEM of *n* = 6. **d** Representative immunoblotting showing overexpression of α-syn in SH-SY5Y cells. α-Tubulin was used as control. **e** Empty vector (pHRIG) (*A*), WT (*B*) or A30P (*C*) α-syn-transduced cells express the same amount of TNFR1 (in *red*). The nuclei are stained with DAPI

After 15 days of culture, the glial cells (Fig. 1a), consisting of astrocytes (GFAP^+^) and microglia (CD68^+^) but free of neurons (MAP2), were challenged with LPS. The medium was collected and ELISA assay was performed in order to observe the presence of the cytokines (TNF-α and IL-1β) and chemokine (KC) (Fig. 1c). To confirm the presence of TNF-α receptor 1 in all groups, we performed an immunofluorescence (Fig. 1e). In order to study the role of CM from activated glia treated with LPS in SH-SY5Y with human α-syn expression, we observed cell viability. Analysis of the LDH release was carried out over the 24-h treatment with CM. Based on the data reported in Fig. 1b, we used 320 μL of CM (EC50) for 5 × 10^4^ cells in subsequent studies, to observe the influence of WT and A30P α-syn overexpression on cell death.

### CM-induced cell death is not affected by NaSal treatment in WT and A30P α-syn

**Fig. 2:**
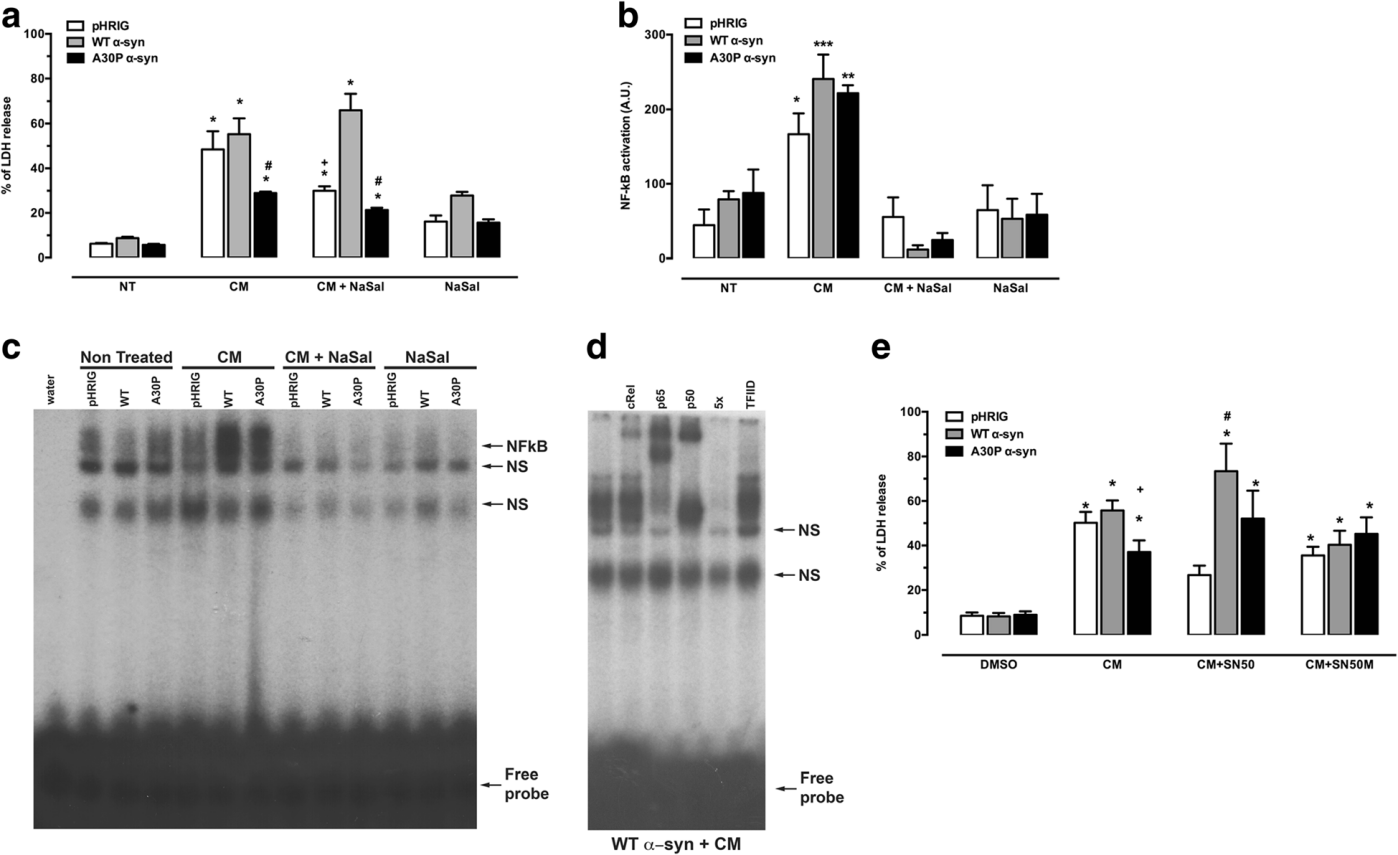
Blockage of NF-κB does not rescue cell death caused by CM in WT or A30P a-syn overexpressing cells. **a** LDH release 24 h after CM treatment. Graph shows mean ± SEM of six independent experiments. The results are expressed as a percentage of maximal LDH release obtained after complete cell lysis. Two-way ANOVA followed by Bonferroni test: *P < 0.05 vs non-treated cells; ^#^ *P* < 0.05 vs WT α-syn treated with CM or CM + NaSal; ^+^ *P* < 0.05 vs pHRIG treated with CM. **b** Densitometry analysis of p65/p50 heterodimers of the nuclear extracts of SH-SY5Y cells. The data represent the mean ± SEM of five independent experiments. Two-way ANOVA followed with Tukey test: *P < 0.05, **P < 0.01 and ***P < 0.001 in NT, CM + NaSal and NaSal only treated cells. **c** Representative EMSA autoradiography. The NF-κB-specific band is indicated by an *arrow. NS* represents non-specific binding. **d** Supershift and competition assay were performed on nuclear extract of SH-SY5Y overexpressing WT α-syn. First lane (from *left* to *right*) represents the WT α-syn treated with CM. Fifth lane represents the presence of unlabelled specific oligonucleotides (NF-κB consensus sequence, fivefold molar excess). Lane 6 represents the presence of non-specific oligonucleotide (TFIID consensus sequence at fivefold molar excess). Supershift assay was performed in the absence and presence of antibodies against NF-κB subunits p65, p50 and cRel, as indicated. **e** Cells treated with CM present with significant cell death compared to DMSO-treated groups (*P < 0.05 vs DMSO). There is no reduction in cell death (WT and A30P) after treatment with SN50 concomitantly with CM. Two-way ANOVA followed with Sidak test: ^#^ *P* < 0.05 vs WT α-syn + CM, ^+^ *P* < 0.05 vs WT α-syn + CM. Graph shows mean ± SEM of four to eight independent experiments

Identification of α-syn overexpression in SH-SY5Y cells transduced with lentivirus pWT α-synIG (WT α-syn) or pA30P α-synIG (A30P α-syn) in comparison to cells transduced with empty vector (pHRIG) is shown in Fig. 1d. As we detected high levels of TNF-α in the CM, we evaluated the expression of TNFR1 on the cells transduced with both variants of α-syn, but there was no difference in TNFR1 levels between the groups (Fig. 1e). To investigate the role of CM produced by activated glia on α-syn overexpressing cells, cells were treated with CM (320 μL/5 × 10^4^ cells) for 24 h. A substantial increase in the percentage of cell death was observed in pHRIG, WT α-syn and A30P α-syn cells when compared to non-treated cells (Fig. 2a). However, A30P α-syn cells treated with CM presented significantly lower LDH release when compared to pHRIG or WT α-syn cells treated with CM. To investigate whether the NF-κB signalling pathway was involved in the cell death caused by CM, SH-SY5Y cells were treated with NaSal, a NF-κB inhibitor. This transcription factor is kept inactive in the cytoplasm via binding to IκB, which, when phosphorylated, releases NF-κB, which then translocates to the nucleus. NaSal inhibits the degradation of IκB, thereby blocking N-κB activation [33]. The cells were treated with CM (320 μL/5 × 10^4^ cells) in the presence or absence of NaSal 10 mM [34] for 24 h. As shown in Fig. 2a, the CM-mediated cell death was reverted by NaSal treatment only in the control group pHRIG but not in WT or A30P α-syn, even though there was significantly less cell death in A30P α-syn treated with CM when compared to WT α-syn treated with CM.

To investigate the involvement of NF-κB in this process, we performed an EMSA, observing that the DNA binding activity to the NF-κB consensus sequence in cells treated with CM was increased in comparison with non-treated (NT) cells (Fig. 2b, c). The simultaneous incubation of CM and NaSal significantly decreased this binding activity. The subunits involved in this binding were p65, cRel and p50 (Fig. 2d), for both WT and A30P α-syn.

To study whether the p50 subunit of NF-κB participates in this protection from cell death triggered by CM, we treated the cells with the inhibitory peptide SN50 (18 μM). As a control, an inactive peptide, SN50M (18 μM), was used. The control cells, pHRIG, treated with CM and SN50 showed a significant decrease in cell death versus cells treated with CM only. No significant difference was observed on cell death in A30P α-syn group challenged with CM and treated with or without SN50. However, there was an increase of LDH release in WT α-syn cells treated with CM and SN50, when compared with WT α-syn treated with CM alone (Fig. 2e).

### Blockage of p42/44 MAPK but not NF-κB abrogates TNF-α-induced cell death

**Fig. 3:**
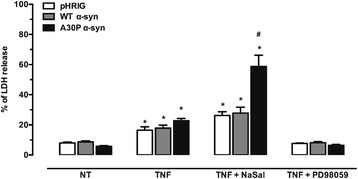
Blockage of p42/44 MAPK but not NF-κB abrogates cell death caused by TNF-α. TNF-α causes significant cell death after 24 h of incubation (*P < 0.05 vs NT and TNF-α + PD98059), which is abrogated by PD98059. Treatment with NaSal does not prevent this cell death. A30P α-syn treated with TNF-α and NaSal shows a higher level of LDH release (^#^ *P* < 0.05) vs A30P treated only with TNF-α. Graph shows mean ± SEM of six independent experiments. The results are expressed as a percentage of maximal LDH release activity obtained after complete cell lysis

To determine whether the involvement of TNF-α impacts on the cell death caused by CM, cells were treated with the TNF-α at 10 ng/mL. As shown in Fig. 3, TNF-α was capable of causing cell death after 24 h of incubation. The treatment together with NaSal did not protect the cells from death. However, the incubation of PD98059 (50 μM), an inhibitor of MEK1 concomitantly with TNF-α, did prevent cell death.

### MAPK inhibitor PD98059 protects from CM-induced cell death

**Fig. 4:**
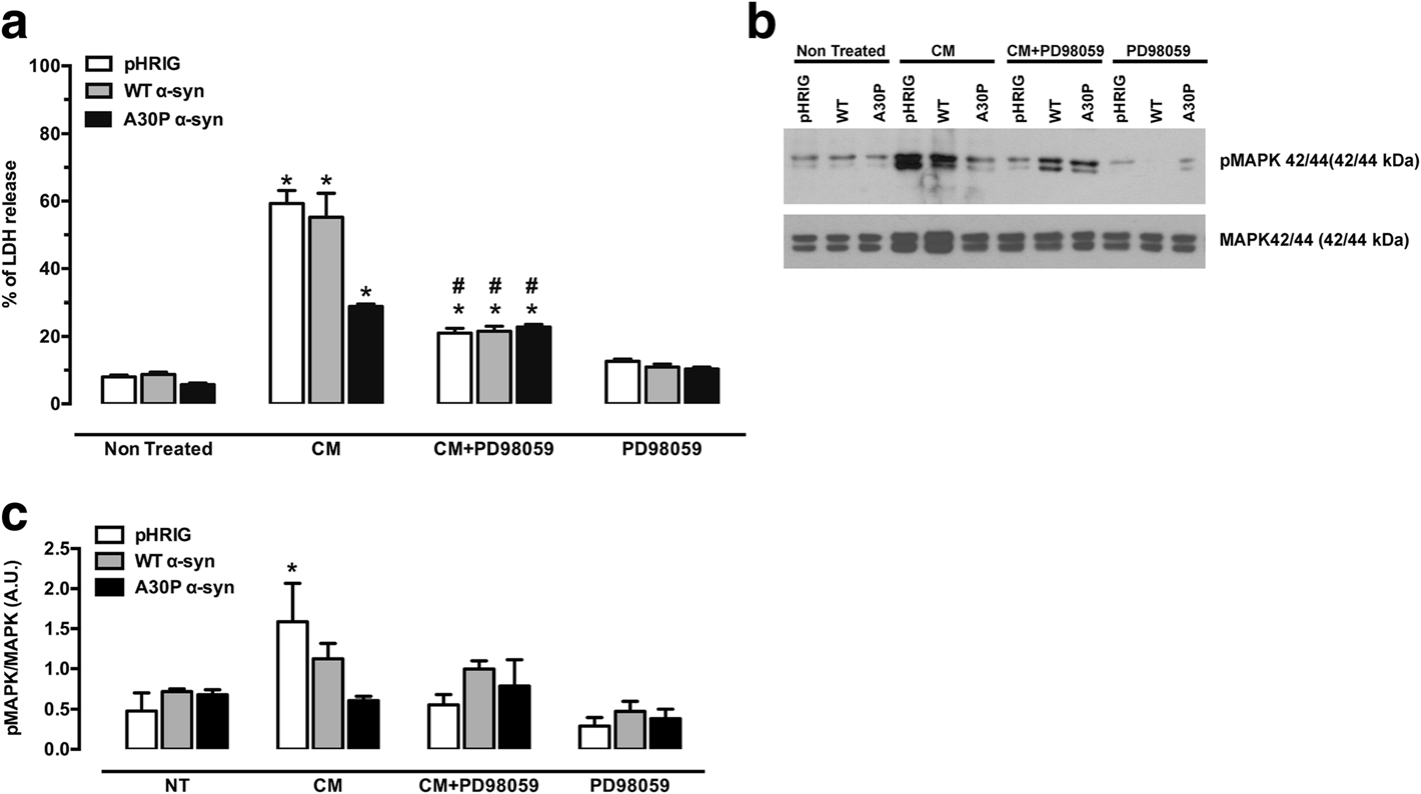
Blockage of p42/44 MAPK protects the SH-SY5Y cells from death caused by CM. **a** CM causes significant cell death after 24 h of incubation (*P < 0.05 vs NT and PD98059), which is decreased by PD98059 (^#^ *P* < 0.05). Graph shows mean ± SEM of five independent experiments. The results are expressed as a percentage of maximal LDH release activity obtained after complete cell lysis. **b** Representative images of phosphorylated and total form of MAPK42/44 immunoblotting assay. **c** The densitometric analysis demonstrates that phosphorylation of MAPK42/44 is significantly higher in pHRIG cells treated with CM versus pHRIG + CM + PD98059. Two-way ANOVA followed with Bonferroni test: *P < 0.05 vs pHRIG + CM + PD98059 and A30P α-syn + CM. Graph shows mean ± SEM of four independent experiments

It is suggested that activation of the MAPK pathway can drive α-syn overexpression [35]. To examine whether MAPK signalling is protective, cells were treated with PD98059, a MEK1 inhibitor (50 μM). After pre-treating the cells with PD98059 for 20 min before the 24-h CM treatment, we detected a decrease of LDH release when compared to CM-treated cells without PD98059 (Fig. 4a). Also, the expression of the phosphorylated form of MAPK42/44 was assayed by immunoblotting (Fig. 4b). We could observe that CM treatment significantly increased the expression of pMAPK42/44 in pHRIG cells but not in WT or A30P α-syn, which was prevented by PD98059 treatment in pHRIG cells (Fig. 4c).

## Discussion

Inflammation is a key process in PD pathogenesis and disease progression. Activated microglia are present in PD animal models and patients, as evidenced in several studies [36, 37]. Microglia and astrocytes can become reactive during PD, releasing cytokines and chemokines [38], with reactive astrocytes being harmful to neurons [37]. Astrocytic CM is also toxic to motor neurons in culture, again increasing cell death and highlighting the glia role in cell death in different neuronal subtypes [39, 40].

Here, we demonstrate that CM activated NF-κB. This can arise as a consequence of the many factors contained in activated glial CM, including several cytokines, chemokines and growth factors that can trigger NF-κB nuclear translocation. In the central nervous system, there is evidence supporting a dual role of NF-κB in neurodegenerative diseases; activation of neuronal NF-κB can promote their survival, whereas NF-κB activation in glial and immune cells mediates pathological inflammatory processes [27]. In this report, we show that the treatment of WT or A30P α-syn cells with CM increases LDH release when compared to cells with no treatment, which is not reverted by blockade of IκB degradation by NaSal or through suppression of the p50 subunit nuclear translocation, suggesting that CM can trigger cell death in a NF-κB-independent manner. Indeed, it has been shown that the interaction between TNF-α and its receptors activates the signalling complex triggering cell death via caspase-8 regulation [41, 42]. In this aspect, we could observe cell death in the presence of TNF-α in all three groups, and no rescue was observed when these cells were co-treated with NaSal, reinforcing the possibility of CM triggering cell death in NF-κB-independent way.

Moreover, the expression of A30P α-syn on neuroblastoma SH-SY5Y decreases cell death triggered by activated glial CM, when compared to WT α-syn or control group. This effect of A30P α-syn may be due to the lower MAPK42/44 phosphorylation when treated with CM, resulting in reduced cell death in comparison with WT α-syn. Furthermore, our data showed that MEK1 inhibition by PD98059 decreases LDH release by CM in SH-SY5Y cells. Recently, Lazaro et al. [43] demonstrated that the A30P mutant showed a reduced propensity to form inclusions when compared to other familial mutations like E46K, which could explain our results in which cells overexpressing A30P α-syn show less cell death compared to WT α-syn cells treated with CM.

Convincing evidence shows a protective role for NF-κB in apoptosis [44]. In fact, we observed that the blockage of NF-κB activation in the SH-SY5Y cells after treatment with TNF-α makes the cells more susceptible to apoptosis, specially in A30P α-syn cells, suggesting that this mutation is more prone to apoptosis when NF-κB is blocked. This suggests that the lysosomal dysfunction [45] could play a role in this process, since LAMP-1 1 (lysosomal-associated membrane protein-1) in A30P cells is less expressed; therefore this group is more sensitive to exposure to TNF-α, which is known to cause disruption of lysosomes [46].

Furthermore, when the p50 subunit of NF-κB is inhibited, there is no decrease in LDH release in WT or A30P α-syn treated with CM, despite the significant reduction in the pHRIG group when co-treated with SN50. Another possibility would be a role for the p65 subunit. The inhibition of p65 activation decreases the neurodegeneration in mice treated with MPTP [24], suggesting that p65 activation is an important event in this PD model [47].

## Conclusions

In conclusion, our results suggest that SH-SY5Y cells transduced with α-syn (WT or A30P) and treated with CM from LPS-activated glia cells show evidence of cell death, which is not reverted by NF-κB blockage by NaSal or SN50. Additionally, the expression of A30P α-syn on neuroblastoma SH-SY5Y leads to decreased cell death triggered by the CM of activated glia, when compared to WT α-syn or control group. The mechanism underlying this process remains to be completely elucidated, but the present data suggest that the inhibition of MEK1 plays an important role in this process.
